# High HER2 protein levels correlate with increased survival in breast cancer patients treated with anti‐HER2 therapy

**DOI:** 10.1016/j.molonc.2015.09.002

**Published:** 2015-09-15

**Authors:** Paolo Nuciforo, Sheeno Thyparambil, Claudia Aura, Ana Garrido-Castro, Marta Vilaro, Vicente Peg, José Jimenez, Rocio Vicario, Fabiola Cecchi, William Hoos, Jon Burrows, Todd Hembrough, Juan Carles Ferreres, José Perez-Garcia, Joaquin Arribas, Javier Cortes, Maurizio Scaltriti

**Affiliations:** ^1^ Molecular Oncology Laboratory, Vall d'Hebron Institute of Oncology, Passeig Vall d'Hebron 119-129, 08035 Barcelona, Spain; ^2^ Universitat Autònoma de Barcelona, Plaça Cívica, 08193 Bellaterra (Cerdanyola del Vallès), Spain; ^3^ OncoPlex Diagnostics (Division of NantOmics, LLC), 9600 Medical Center Drive, Suite 300, Rockville, MD 20850, USA; ^4^ Department of Oncology, Vall d'Hebron University Hospital, Vall d'Hebron Institute of Oncology, Passeig Vall d'Hebron 119-129, 08035 Barcelona, Spain; ^5^ Department of Pathology, Vall d'Hebron University Hospital, Passeig Vall d'Hebron 119-129, 08035 Barcelona, Spain; ^6^ Preclinical Research Program, Vall d'Hebron Institute of Oncology, Passeig Vall d'Hebron 119-129, 08035 Barcelona, Spain; ^7^ Institució Catalana de Recerca i Estudis Avançats (ICREA), 08010 Barcelona, Spain; ^8^ Human Oncology & Pathogenesis Program (HOPP), Memorial Sloan-Kettering Cancer Center, 1275 York Avenue, Box 20, New York, NY 10065, USA

**Keywords:** Breast cancer, HER2, Mass spectrometry, Trastuzumab, Immunohistochemistry, In situ hybridization

## Abstract

*Introduction:* Current methods to determine HER2 (human epidermal growth factor receptor 2) status are affected by reproducibility issues and do not reliably predict benefit from anti‐HER2 therapy. Quantitative measurement of HER2 may more accurately identify breast cancer (BC) patients who will respond to anti‐HER2 treatments.

*Methods:* Using selected reaction monitoring mass spectrometry (SRM‐MS), we quantified HER2 protein levels in formalin‐fixed, paraffin‐embedded (FFPE) tissue samples that had been classified as HER2 0, 1+, 2+ or 3+ by immunohistochemistry (IHC). Receiver operator curve (ROC) analysis was conducted to obtain optimal HER2 protein expression thresholds predictive of HER2 status (by standard IHC or in situ hybridization [ISH]) and of survival benefit after anti‐HER2 therapy.

*Results:* Absolute HER2 amol/μg levels were significantly correlated with both HER2 IHC and amplification status by ISH (p < 0.0001). A HER2 threshold of 740 amol/μg showed an agreement rate of 94% with IHC and ISH standard HER2 testing (p < 0.0001). Discordant cases (SRM‐MS‐negative/ISH‐positive) showed a characteristic amplification pattern known as double minutes. HER2 levels >2200 amol/μg were significantly associated with longer disease‐free survival (DFS) and overall survival (OS) in an adjuvant setting and with longer OS in a metastatic setting.

*Conclusion:* Quantitative HER2 measurement by SRM‐MS is superior to IHC and ISH in predicting outcome after treatment with anti‐HER2 therapy.

## Highlights

- We quantified by mass spectrometry the levels of HER2 in HER2‐positive breast tumors.
- The tumors with the highest HER2 levels benefitted most from HER2 inhibition.
- HER2 protein levels were better predicting markers than gene amplification.
- Double minute amplification pattern was associated with low HER2 levels.

**Abbreviations**
HER2: human epidermal growth factor receptor 2
SRM-MS: selected reaction monitoring mass spectrometry
ROC: receiver operating characteristic
MS: mass spectrometry
CEP17: centromere 17
DM: double minutes
HSR: homogeneously staining regions
MIX: mixed
GCN: gene copy number
HR: hormone receptor
ADCC: antibody-dependent cell cytotoxicity

## 1 Introduction

Gene amplification or protein overexpression of the human epidermal growth factor receptor type 2 (HER2) has been reported in ∼20% of invasive breast cancer (BC) and is usually associated with worse prognosis (Slamon et al., 1987, 1989). The monoclonal antibody trastuzumab has dramatically increased survival in patients with HER2‐overexpressing metastatic disease (Hudis, 2007; Slamon et al., 2001) and has often proved curative when used in combination with chemotherapy in the adjuvant setting (Joensuu et al., 2006; Piccart‐Gebhart et al., 2005; Romond et al., 2005; Smith et al., 2007).

The benchmarks for defining tumors as HER2‐positive (HER2+) is the presence of protein overexpression (3+) by immunohistochemistry (IHC) or gene amplification by in situ hybridization (ISH), according to current clinical guidelines (Wolff et al., 2013). However, considerable controversy still exists regarding the accuracy, reliability, and inter‐observer variability of these methods. Studies in patients treated with trastuzumab indicate that neither test is a perfect predictor of response to trastuzumab (Dowsett et al., 2009; Perez et al., 2010). It is estimated that up to 20% of tumors initially classified as HER2+ by IHC are actually false‐positives (Paik et al., 2002; Perez et al., 2006; Roche et al., 2002), and an estimated 1.1%–11.5% of HER2‐negative (HER2‐) patients by IHC that never received anti‐HER2 therapy harbor HER2 gene amplification by ISH (Hanna et al., 2014). False positives HER2 results increase treatment costs (trastuzumab costs $50,000/person/year in the US) and expose patients to a likely ineffective therapy; false negative results deny patients the potential benefits of anti‐HER2 therapy.

It is widely accepted that the levels of HER2 are not homogeneous among the HER2+ population defined by conventional semi‐quantitative methods such as IHC. Tests capable of absolute quantitation of HER‐family protein expression have demonstrated that HER2 protein expression can vary up to 100 fold and that tumors with high HER2 expression are more likely to benefit from anti‐HER2 therapy in the neoadjuvant (Cheng et al., 2014; Denkert et al., 2013), adjuvant (Pogue‐Geile et al., 2013), and metastatic (Montemurro et al., 2014) settings.

We have recently developed a mass spectrometry (MS)‐based proteomic BC panel to measure the absolute abundance of targeted proteins in patient‐derived formalin fixed, paraffin embedded (FFPE) tissue for use in clinical decision‐making. The reliability of this assay for protein analysis has been demonstrated (Hembrough et al., 2012), however, its clinical utility for patient stratification, choice of therapy, and drug resistance prediction is still being evaluated. In this work, we tested this methodology's ability to predict HER2 status as determined by standard IHC/ISH in a panel of breast tumors. We also assessed the value HER2 quantitation by MS for predicting disease‐free survival (DFS) or overall survival (OS) of patients with HER2‐positive BC after treatment with anti‐HER2 therapy.

## 2 Material and methods

### 2.1 Patients and tissue samples

Samples of histologically confirmed invasive BC diagnosed at Vall d'Hebron University Hospital (Barcelona, Spain) were retrospectively identified by one study pathologist (CA) between 1997 and 2013. Samples were selected to ensure a representative number of HER2‐ and HER2+ samples and to include cases treated with trastuzumab to enable survival analyses in a subset of patients. Sample selection criteria were: known HER2 status tested in the setting of the routine surgical pathology laboratory and available FFPE tumor sample for SRM‐MS analysis. For survival analyses, samples with available data on type of anti‐HER2 treatment and outcome were included. The study was approved by the hospital ethical committee, including a waiver of consent for the use of archival material for research.

### 2.2 HER2 standard testing (combined IHC/ISH)

HER2 status was retrieved from hospital Vall d'Hebron pathology laboratory reports (HER2 local). The diagnostic algorithm for HER2 testing used was IHC on all cases and ISH assays done on all IHC2+ equivocal cases (per ASCO/CAP guidelines). Protein expression was determined in paraffin‐embedded sections using the 4B5 (Ventana Medical Systems, Tucson, AZ) antibody. *HER2* amplification was determined using silver‐enhanced ISH (SISH) and carried out with an INFORM HER2 Dual ISH DNA Probe Assay (Ventana). Testing was performed and scored according to both the 2007 ASCO/CAP guidelines and the 2013 update of these guidelines.

For the purpose of this study, and to exclude any possibility of heterogeneity in the tumor with respect to HER2 expression, IHC and ISH were repeated on all HER2 3+ patients with available tissue samples and on discordant cases on the same tissue block of the resection specimen sent for SRM‐MS testing (HER2 central). Central testing was performed using IHC (HercepTest) and FISH (HER2 FISH pharmDx™ Kit) or SISH (INFORM HER2 Dual ISH DNA Probe Assay, Ventana) according to ISO15189 standards and interpreted following the most recent ASCO/CAP guidelines. *HER2* gene status was assessed by two pathologists (CA and VP) blindly scoring 30 nuclei for the number of *HER2* and *centromere 17* (*CEP17*) signals in each cell. The *HER2/CEP17* probe signal ratio was determined and the patterns of *HER2* amplification were analyzed in those cases with *HER2/CEP17* ratio ≥2. Samples with >70% of the cells with double minutes (DM, small dispersed dots distributed through the nucleus) or homogeneously staining regions (HSR, tightly clustered dots in discrete regions of the nucleus) patterns were classified accordingly. Cases with both HSR and DM patterns in the same sample were classified as mixed (MIX).

### 2.3 HER2 quantification by SRM‐MS

HER2 protein was quantitated by SRM‐MS as previously described (Hembrough et al., 2013). Briefly, tissue sections (10 μM) were cut from FFPE blocks, placed onto DIRECTOR® microdissection slides, deparaffinized and stained with hematoxylin. Tumor areas were marked by a board‐certified pathologist and a cumulative area of a 12 mm^2^ (from multiple sections of a single tumor if necessary) containing approximately 45,000 malignant cells was microdissected from each tumor and then solubilized to tryptic peptides using Liquid Tissue® technology. This tryptic peptide mixture was then subjected to SRM‐MS analysis using stable isotope‐labeled internal standard for accurate quantitation of analytical targets. The peptide that was chosen for HER2 was ELVSEFSR (located in the intracellular region of HER2, aa 971–978). This peptide is unique to HER2 and has been reported to be the best sequence for SRM in FFPE tissue (Schoenherr et al., 2012; Steiner et al., 2015). On‐column injection resulted in 1 μg (∼4000 cells) of solubilized tissue and 5 fmol of internal standard measured by microBCA (ThermoFisher Scientific, San Jose, CA). Instrumental analyses were performed on TSQ series (Vantage or Quantiva) triple quadrupole mass spectrometer (Thermo Scientific, San Jose, CA). The MS and chromatography conditions have been previously described (Catenacci et al., 2014).

### 2.4 Statistical methods

To select a SRM‐MS threshold for stratifying tumors into HER2+ and HER2‐, receiver operating characteristic (ROC) curves were constructed by computing the sensitivity and specificity of increasing quantities of HER2 (by SRM‐MS) in predicting HER2 positivity (by combined IHC/ISH). Differences in continuous HER2 measurements among various IHC and ISH subgroups were analyzed by using Kruskal–Wallis tests. The Spearman rank correlation coefficient (Spearman ρ) was used to describe the relationship between the HER2 protein levels by SRM‐MS, *HER2* gene copy number (GCN) and *HER2/CEP17* ratio by central ISH. Among patients who had received anti‐HER2 therapy, ROC analysis was used to establish an optimal cutoff for HER2 levels (by SRM‐MS, *HER2/CEP17* ratio and *HER2* GCN) that would predict disease‐free, progression‐free, and overall survival in the adjuvant and metastatic settings. Chi‐square test and Fisher's exact test were used to determine the nature of the associations between optimal cutoff points and clinicopathological parameters. Survival was modeled using the Kaplan–Meier curves, and the significance of differences between these curves was determined using hazard ratio (HR) and its confidence interval of 95%, and the p‐value obtained by the log‐rank test. Multivariate survival analysis was performed using the Cox proportional hazards model adjusted for hormone receptor status, tumor stage, lymph node status and HER2 SRM levels. Results were considered significant when p‐values (*p*) were less than 0.05. Statistical analyses were conducted using R software, version 3.0.3.

## 3 Results

### 3.1 Patients and tissue samples

**Figure 1:**
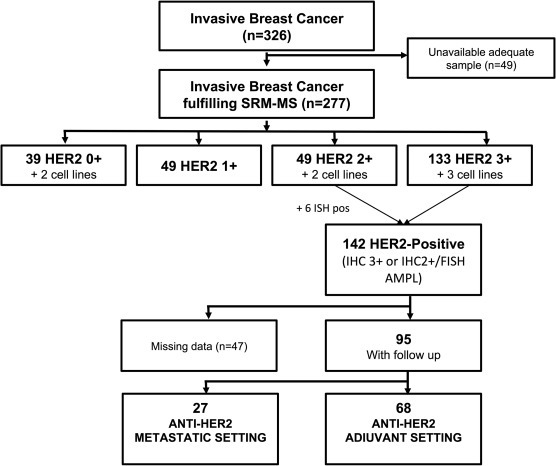
Breast cancer tumor samples selected for analysis. SRM, selected reaction monitoring; IHC, immunohistochemistry; ISH, in situ hybridization; AMPL, amplified.

We identified 326 samples, of which 277 (85%) were suitable for SRM‐MS analysis. Forty‐nine samples were discarded for lack of sufficient tumor tissue for SRM‐MS. Of the 277 study samples, 270 were FFPE breast cancer samples and 7 were cell lines (Figure 1, Table 1 and Supplementary Table 1). Patient specimens were obtained mainly from surgical resection (n = 255), and a small part from diagnostic core biopsies (n = 6) or sampling of recurrent disease (n = 9). The study series included 41 HER2 0+, 49 HER2 1+, 51 HER2 2+, and 136 HER2 3+ assessed by IHC. Of the 142 samples classified as HER2+ by combined IHC/ISH approach, 95 were included in the survival analysis (Supplementary Table 2). Forty‐seven were excluded due to the following: twenty‐five were replicated samples from the same patients; seven were lost to follow up; five were still under treatment at the time of the analysis; three received trastuzumab after 12 months from diagnosis (atypical adjuvant); three were cell lines; two had a bilateral invasive breast carcinoma; and two had received trastuzumab as neoadjuvant treatment. Sixty‐eight patients received adjuvant chemotherapy in combination with trastuzumab alone (76%, n = 52) or combined with another anti‐HER2 agent (24%, lapatinib, n = 6 and pertuzumab, n = 10). Twenty‐seven received anti‐HER2 therapy in the metastatic setting. Trastuzumab alone was the preferred anti‐HER2 treatment (70%, n = 19), followed by trastuzumab combined with another anti‐HER2 (22%, pertuzumab, n = 5; lapatinib, n = 1), T‐DM1 (4%, n = 1), and T‐DM1 plus pertuzumab (4%, n = 1).

**Table Table 1:** Characteristics of 270 clinical samples used in the study.

Characteristics	N°	%
Patient specimen		
Surgical resection	255	95
Diagnostic core biopsy	6	2
Recurrent disease	9	3
Histological grade		
G1	16	6
G2	112	41
G3	134	50
Unknown	8	3
Pathological stage T		
Tx–T1	143	53
T2–T4	123	46
Unknown	4	1
Pathological stage N		
Nx–N0	147	54
N1–N3	119	44
Unknown	4	1
Hormone receptor status (HR)		
Negative	47	17
Positive	223	83
HER2 overexpression by IHC		
0	39	14
1	49	18
2	49	18
3	133	49

### 3.2 SRM‐MS versus standard IHC/ISH

The average HER2 protein level in the analyzed dataset (n = 277) as measured by SRM‐MS was 2217.9 amol/μg (median: 643.5; sd: 3299.4; range: 0 to 17,446.7). Absolute HER2 amol/μg levels increased with increasing IHC scores with averages values of 189.1, 259.9, 406.7 and 4214.1 in HER2 IHC 0+, 1+, 2+, and 3+, respectively (p < 0.001, Kruskal–Wallis rank sum test) (Supplementary Table 3). Samples scored as HER2 IHC3+ expressed the widest dynamic range of HER2 protein levels as quantified by SRM‐MS (range: 163.7–17,446.7 amol/μg). When correlated with amplification status, HER2 protein levels were also substantially higher in ISH‐amplified (mean: 4151.2 amol/μg; sd: 3682.1; range: 272.8–17,446.7) than non‐amplified samples (mean: 383.8 amol/μg; sd: 339.1; range: 0–1748.0; p < 0.001, Wilcoxon test, Supplementary Table 4). In our study we analyzed samples collected from 1997 to 2013. Although the SRM‐MS is an epitope‐independent technology and its robustness has been proven (Catenacci et al., 2014), we addressed the stability of HER2 as detected by SRM‐MS over time. The average SRM‐MS values did not differ significantly with age of the tissue blocks thus supporting the validity of results generated using samples collected over a period of many years (Supplementary Table 5).

**Figure 2:**
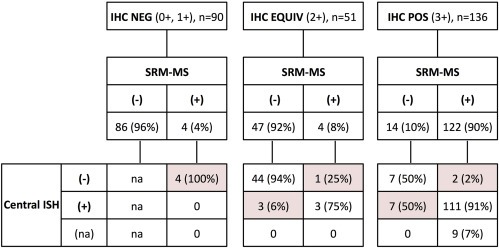
HER2 selected reaction monitoring‐mass spectrometry (SRM‐MS) results for 277 breast cancers previously classified as IHC negative (0+, 1+), equivocal (2+) or positive (3+) with subsequent ISH central retesting results. SRM‐MS‐positive = HER2 protein >740 amol/μg. Discordant cases are highlighted. −, negative; +, positive; na, not assessed.

Per ROC analysis, the SRM‐MS threshold that best correlated with HER2 status by combined local IHC/ISH was 740 amol/μg (area under the ROC curve: 0.963). When stratified according to this threshold, 130 samples (47%) were classified as overexpressors and 147 (53%) as non‐overexpressors. The overall percent agreement between SRM‐MS and combined local IHC/ISH was 92% (255 of 277). The percent positive agreement was 88% (125 of 142), and the percent negative agreement was 96% (130 of 135) (Table 2). HER2 status by SRM‐MS for 277 breast cancer samples that had been previously scored by local IHC testing and subsequently evaluated by ISH reflex central testing are shown in Figure 2. In the HER2 IHC negative group (0+ and 1+, n = 90), 86 samples (96%) were correctly classified as negative and 4 (4%) as positive by SRM‐MS. After central retest, none of these 4 positive samples showed *HER2* amplification. In the HER2 IHC equivocal group (2+, n = 51), 47 (92%) and 4 (8%) samples were classified as negative and positive by SRM‐MS, respectively. Three of these 4 SRM‐MS‐positive samples were *HER2* amplified (the non‐amplified discordant sample was the ZR75‐1 cell line). Three out of 47 samples (6%) classified as negative by SRM‐MS showed *HER2* gene amplification.

**Table Table 2:** Concordance between SRM‐MS and local and central combined IHC/ISH.

	HER2 Status (IHC/ISH)	n	SRM‐MS Agreement, n (%)
Local	Negative	135	130 (96%)
	Positive	142	125 (88%)
Central	Negative	144	137 (95%)
	Positive	133	123 (92%)

Local, local HER2 testing result; Central, central HER2 re‐testing results; SRM‐MS, selected reaction monitoring mass spectrometry. IHC, Immunohistochemistry; ISH, in situ hybridization.

In the HER2 IHC positive group (3+, n = 136), 122 samples (90%) were correctly classified as positive whereas 14 samples (10%) as negative by SRM‐MS. *HER2* gene amplification was centrally confirmed in 111 (98%) of the 113 evaluable IHC3+/SRM‐MS‐positive samples (not amplified, n = 2; data not available, n = 9). Among IHC3+/SRM‐MS‐negative (n = 14), seven harbored *HER2* gene amplification. Overall agreement after central retest was 94% (260 of 277), the percent positive agreement was 92% (123 of 133), and the percent negative agreement was 95% (137 of 144) (Table 2). Details of the discordant samples between SRM‐MS and combined IHC/ISH are shown in Supplementary Table 6.

### 3.3 Relationship between *HER2* gene amplification pattern and HER2 protein levels

After central retest, 6% (17/277) of samples remained discordant. The 7 SRM‐MS‐positive/ISH‐negative samples showed absolute HER2 protein levels below the average dataset value (2217.9 amol/μg) and very close to the 740 amol/μg threshold distinguishing overexpressors from non‐overexpressors. The remaining 10 samples showed low protein levels (<740 amol/μg) despite *HER2* gene amplification. When stratified by *HER2* amplification pattern, 8 of 10 samples had patterns involving extrachromosomal circles of DNA known as DM and the remaining 2 showed a mixed amplification pattern. No significant differences in *HER2/CEP17* ratios were evident (data not shown).

**Figure 3:**
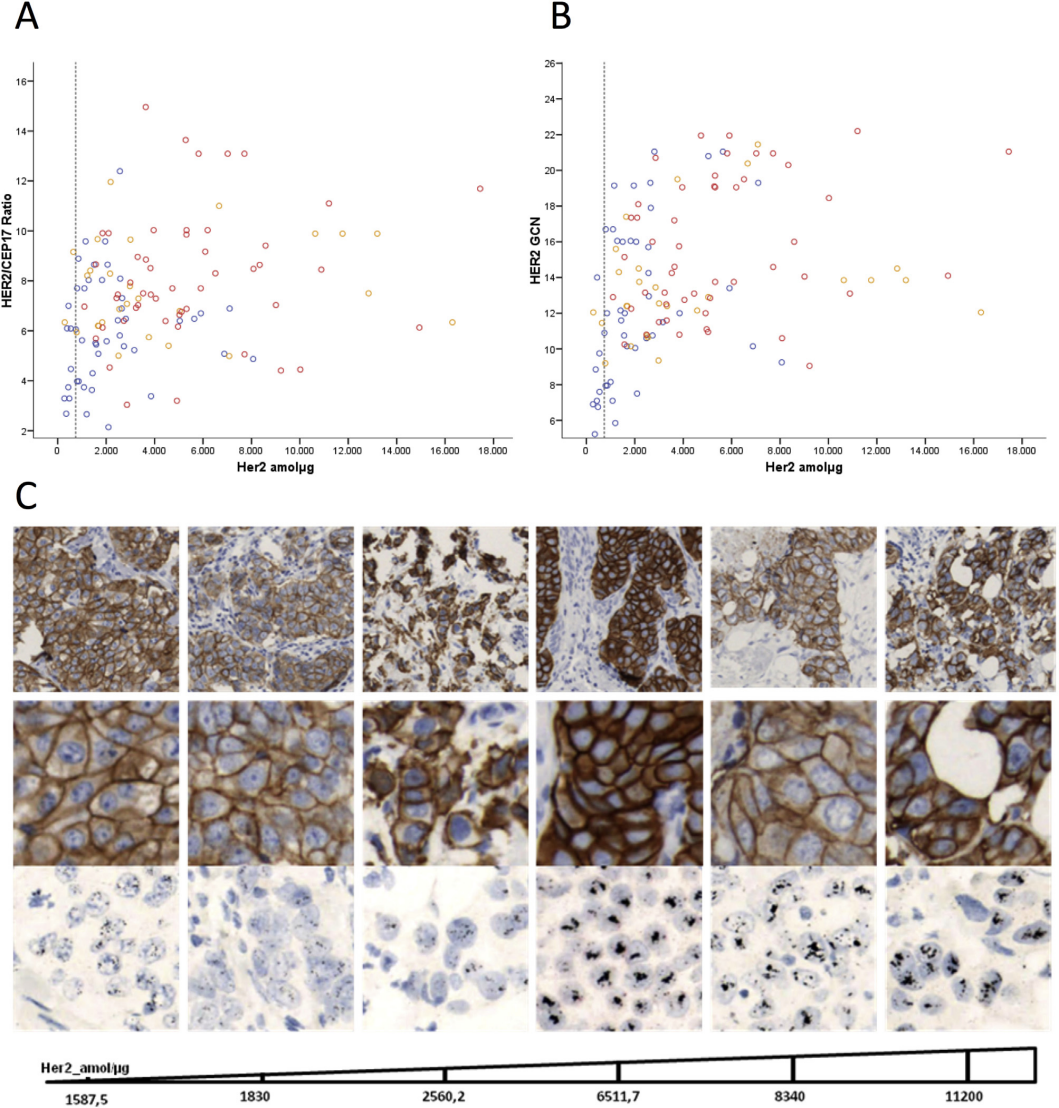
Correlation between the HER2 protein expression by SRM‐MS and HER2/CEP17 ratio (A), and HER2 GCN (B). Dotted gray line indicates HER2 SRM‐MS 740 amol/μg threshold. Spearman rank correlation coefficient was used to describe the relationship between SRM‐MS and HER2 GCN (ρ, 0.44; p < 0.001) and SRM‐MS and HER2/CEP17 (ρ, 0.31; p < 0.001). Pattern of amplification by in situ hybridization is shown. Red circle, homogeneously staining regions (HSR); blue circle, double minutes (DM); orange circle, Mixed pattern. c, Representative images of protein expression by IHC and amplification patterns by ISH are shown together with SRM‐MS protein levels.

We therefore investigated whether, in the presence of *HER2* gene amplification, the levels of HER2 protein in the tumor tissue may be influenced by its amplification pattern rather than the levels of gene amplification itself. To test this hypothesis, we correlated HER2 expression by SRM‐MS with *HER2* GCN, *HER2/CEP17* ratio and pattern of amplification (HSR, DM, MIX) in HER2 IHC 2+ (n = 6) and IHC3+ (n = 117) cases amplified by central ISH. The mean HER2 protein SRM‐MS level was 4047.1 amol/μg (sd: 3508.9; range: 272.8–17,446.7). Mean *HER2* GCN was 14.0 (sd: 4.2; range: 5.2–22.2). Mean *HER2/CEP17* ratio was 7.2 (sd: 2.5; range: 2.1–15.0) (Supplementary Table 7). HER2 SRM‐MS levels showed weak positive correlations with *HER2* GCN (Spearman ρ = 0.44; p < 0.001) and *HER2/CEP17* ratio (Spearman ρ = 0.31; p < 0.001) (Figure 3). Forty‐one percent of samples (n = 50) had HSR patterns, 37% (n = 46) had DM patterns and 22% (n = 27) were mixed. Average HER2 protein levels were significantly higher in tumors amplified with HSR (mean: 5462.9; sd: 3368.4; range: 1099.3–17,446.7) compared to those with DM (mean: 2176.4; sd: 1908.1; range: 272.8–8070.0) (Supplementary Table 8).

DM amplification patterns were present in 80% (8/10) of samples with low HER2 protein levels; only 34% (38/113) of samples with high HER2 protein levels had DM patterns (Supplementary Table 9).

### 3.4 Survival analyses

**Figure 4:**
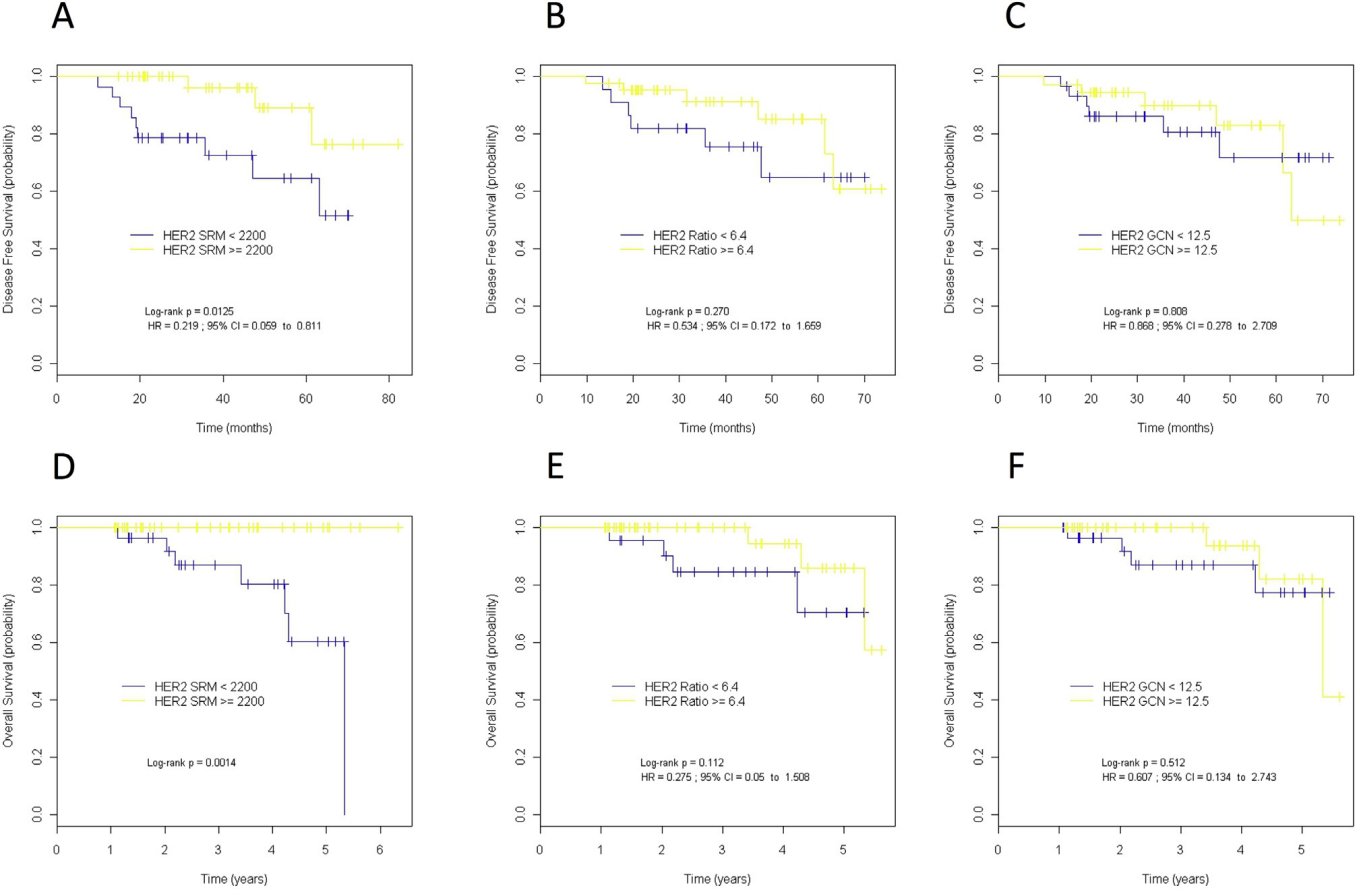
Kaplan–Meier curves for disease‐free survival (A–C) and overall survival (D–F) according to HER2 protein expression by SRM‐MS (A,D), HER2/CEP17 ratio (B,E) and HER2 gene copy number (GCN) (C,F) in patients treated with anti‐HER2 in the adjuvant setting. Optimal cutoff values were determined by receiver operating characteristic (ROC) analysis. Disease‐free survival and overall survival were superior for the group of patients with high HER protein levels (>2200). HR, hazard ratio; HER2 Ratio, HER2/CEP17 ratio; CI, confidence interval.

ROC analysis of patients treated with anti‐HER2 therapy (n = 95) resulted in cutoff values of 2200 amol/μg for HER2 SRM‐MS, 6.4 for *HER2/CEP17* ratio and 12.5 for *HER2* GCN (Supplementary Table 10). The 2200 amol/μg HER2 SRM‐MS cutoff outperformed the 740 cutoff in predicting DFS and OS (Supplementary Table 11) and was used for survival analyses. The correlations between the optimal HER2 SRM‐MS, *HER2/CEP17* ratio and *HER2* GCN cutoffs for survival and clinic–pathological parameters are shown in Supplementary Table 12 and 13. Patients showing HER2 levels by SRM‐MS above the threshold of 2200 amol/μg (n = 58, 61%) were defined as super‐expressors. In the adjuvant setting (n = 68), super‐expressors had a statistically significantly better outcome than non‐super‐expressors (Figure 4). The number of observed DFS events were 3 in the super‐expressors compared to 9 events observed in tumors with HER2 levels below 2200 amol/μg (HR = 0.22, 95% CI 0.06–0.81, log rank p = 0.013). Differences in DFS were even greater between refractory patients (relapse within 24 months) and patients without relapse or recurrent disease within 24 months (OR = 23, 95% CI, 1.26–434.86, p 0.003).

Similar results were observed for OS. None of the super‐expressors died of the disease compared to 7 patients whose tumors were below 2200 amol/μg (HR = na, p 0.001). Neither *HER2/CEP17* ratio nor HER2 GCN was predictive of longer DFS or OS in the adjuvant setting (Figure 4).

**Figure 5:**
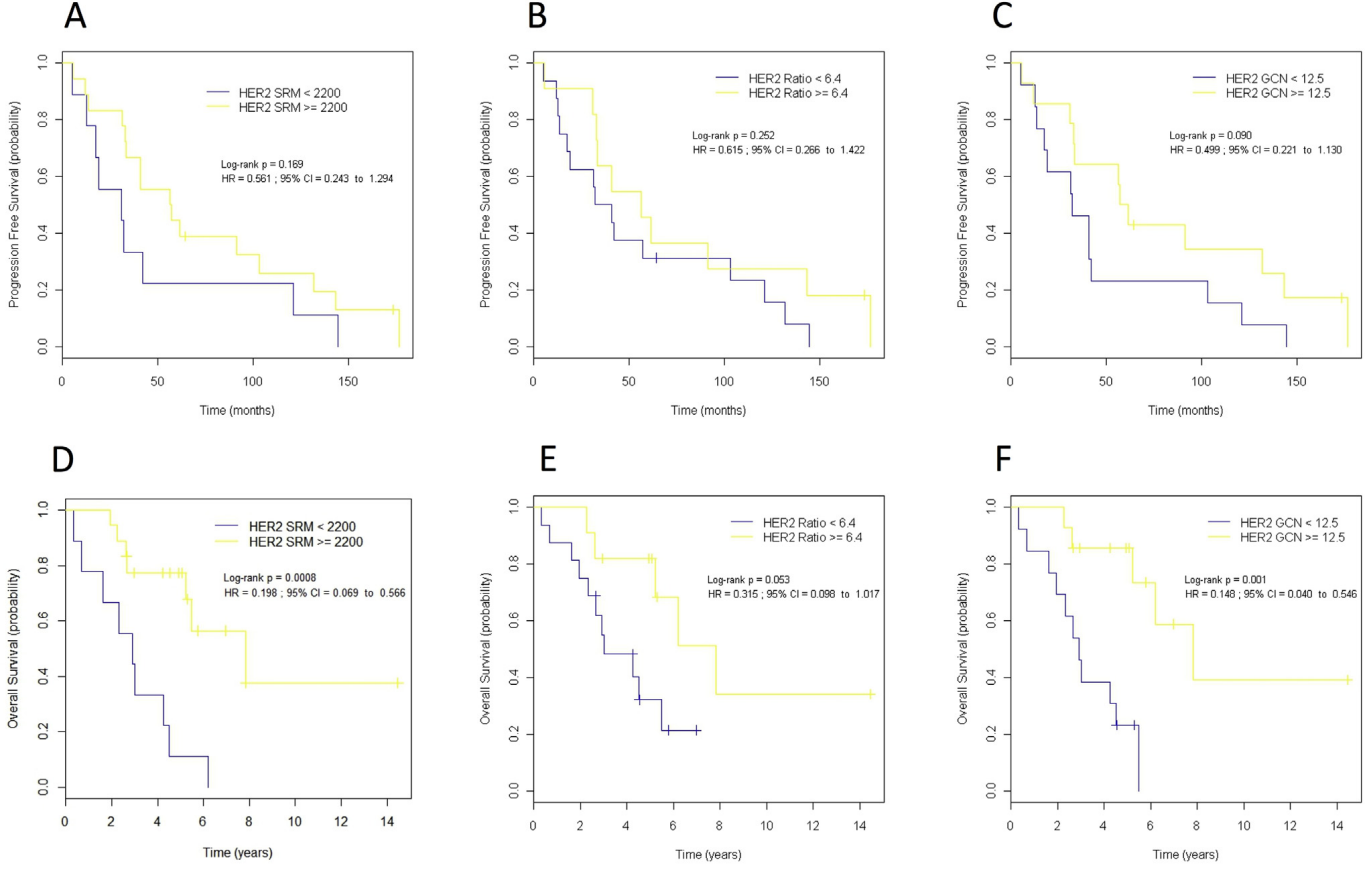
Kaplan–Meier curves for progression‐free survival (A–C) and overall survival (D–F) according to HER2 protein expression by SRM‐MS (A,D), HER2/CEP17 ratio (B,E) and HER2 gene copy number (GCN) (C,F) in patients treated with anti‐HER2 in the metastatic setting. HR, hazard ratio; HER2 Ratio, HER2/CEP17 ratio; CI, confidence interval.

In the first‐line metastatic setting (n = 27), 18 (67%) patients were classified as super‐expressors. Median OS was significantly longer in super‐expressors (7.84; 95% CI: 5.23 to NA) as compared to non‐super‐expressors (2.91; 95% CI: 1.61 to NA), (HR = 0.20; 95% CI: 0.07 to 0.57; p < 0.001), (Figure 5). In this setting, HER2 GCN (HR = 0.15; p = 0.001) and, to a lesser extent, *HER2/CEP17* ratio (HR = 0.32; p = 0.050) were also predictive of a better OS. No significant correlations were found between HER2 protein levels or gene status and PFS, likely due to the fact that all but two patients relapsed during follow up. When looking at relapse within 24 months, nine of 18 (50%) super‐expressors were refractory to anti‐HER2 therapy compared to all (n = 9) patients with HER2 lower than 2200. No correlation was found between HER2 pattern of amplification and survival in both adjuvant and metastatic series (Supplemental Table 14).

In the multivariate model which includes hormone receptor status, tumor size (T) and presence of lymph‐node metastases (N), HER2 levels by SRM‐MS independently predicted DFS in the adjuvant setting (HR = 0.25; 95% CI: 0.06 to 0.96; p = 0.044) (Table 3). The model could not be run for OS due to lack of events in the HER2 super‐expressor group.

**Table Table 3:** Multivariate analyses of disease‐free survival (DFS) in the 68 patients included in the adjuvant series.

DFS	HR	CI95%(HR)	p‐value
$\frac{Hazard\left(\mathrm{Hormone}\;\mathrm{receptor}\;positive\right)}{Hazard\left(\mathrm{Hormone}\;\mathrm{receptor}\;negative\right)}$	0.19	0.05–0.70	0.012
$\frac{Hazard\left(TX-T1\right)}{Hazard\left(T2-T4\right)}$	0.20	0.05–0.73	0.015
$\frac{Hazard\left(NX-N0\right)}{Hazard\left(N1-N3\right)}$	1.19	0.36–3.94	0.777
$\frac{Hazard\left(HER2\geq 2200\right)}{Hazard\left(HER2<2200\right)}$	0.24	0.06–0.96	0.044

## 4 Discussion

This report demonstrates the application of a MS‐based method to objectively quantify HER2 protein in FFPE clinical tissue samples from BC patients. We showed that within IHC‐positive (3+) ISH‐amplified tumors, a wide dynamic range of HER2 protein expression is found and the subgroup of tumors with the highest levels benefitted most from HER2 inhibition. Our findings suggest that quantitative HER2 measurement is superior to gene amplification levels in determining which patient will benefit from trastuzumab treatment in both adjuvant and metastatic settings.

The ASCO/CAP guidelines (Wolff et al., 2013) recommend initial HER2 screening of all BC, followed by ISH for samples with equivocal staining; the results of these tests determine a patient's eligibility for trastuzumab. However, lack of concordance between IHC and ISH (IHC‐negative/ISH‐positive) occurs in up to 11.5% of cases (Hanna et al., 2014). Our findings suggest that these conflicting results may be only marginally due to pre‐analytic (fixation affects antibody sensitivity), analytic (limited dynamic range of chromogenic IHC, different antibodies used), or post‐analytic (subjectivity in interpretation of the results) factors (Camp et al., 2002; Rimm, 2006). In fact, despite the high correlation observed with IHC score or gene amplification detected by ISH, we found that approximately 10% of *HER2*‐amplified breast tumors expressed very low amounts of HER2 protein; all of these discordant cases were associated with a gene amplification pattern known as DM.

Evidence indicates that the amplification of genes in DM may result in a dynamic regulation of gene expression and resistance to EGFR TKIs for EGFR*vIII*‐positive glioblastomas (Nathanson et al., 2014). Conversely, data from our group did not find any significant correlation between amplification of HER2 in DM content and sensitivity to anti‐HER2 therapy (Vicario et al., 2015). Quantitative HER2 protein analysis, however, may identify a subset of HER2 tumors amplified in DM with low HER2 expression that are less sensitive to anti‐HER2 treatment.

Based on our analysis, patients expressing greater than 740 amol/μg of HER2 should receive anti‐HER2 treatment, as this was the optimal threshold that correlated with standard IHC/ISH. However, the most meaningful endpoint of HER2 testing is not prediction of HER2 status by IHC or ISH, but outcome after HER2‐targeted therapies. Using quantitative HER2 measurement, we found that patients whose tumors expressed HER2 protein level >2200 amol/μg) benefitted more from anti‐HER2 therapy than patients with lower HER2 expression levels. Strikingly, relapse within 24 months was observed in 21% of patients with HER2 expression levels below 2200 amol/μg and none of the super‐expressors progressed to therapy in this period of time. One possible explanation is that tumors with high levels of HER2 are enriched with “true” HER2‐dependent disease and therefore potentially more susceptible to HER2 blockade (Montemurro et al., 2014). Another explanation is that the more HER2 receptors are present in the membrane of tumor cells, the more molecules of trastuzumab (or other anti‐HER2 antibodies) can bind and prime antibody‐dependent cell cytotoxicity (ADCC). A direct correlation between HER2 levels and ADCC has been reported in preclinical models (Scaltriti et al., 2009).

Our findings should be considered in light of certain limitations. The number of patients included in this proof‐of‐concept study is small and the cutoff point of 2200 amol/μg was based on the survival outcomes in patients whose tissues were selected for the analysis. This cutoff needs to be validated in a larger, independent set of patients. Also, survival analyses included only individuals who had received anti‐HER2 treatment. Prospective trials will be needed to address the question of whether varying levels of HER2 positivity are truly predictive of response in all BC patients. Studies are underway to validate the cut‐off in an expanded BC cohort.

## 5 Conclusions

HER2 protein quantitation by SRM‐MS in FFPE tissues is predictive of response to anti‐HER2 therapy and survival in HER2‐positive (by standard IHC/FISH) BC patients. Moreover, this methodology may allow the identification of FISH positive cases that express low amounts of HER2 and respond poorly to anti‐HER2 therapy.

## Disclosure statement

ST, WH, TH, FC, and JB are/were paid employees and stock owners at OncoPlexDx, which developed the assay approach described in this report. The remaining authors have no conflict of interests to declare.

## Author agreement

The corresponding author certifies that all authors of this manuscript have seen and approved the version being submitted. The manuscript is the authors' original work, has not received prior publication and is not under consideration for publication elsewhere.

## Authors' contributions

PN, JC, MS, TH, and JB designed the study. ST, CA, AGC, VP, JJ, RV, JCF, JPG, FC, WH, participated in the data collection and generation of results. PN, MS, JC, and MV analyzed data. PN, ST, MS drafted the article. JC, TH, JA revised the article critically for important intellectual content.

## Supporting information



The following is the supplementary data related to this article:

Supplementary data

## Supplementary data 1

Supplementary data related to this article can be found at http://dx.doi.org/10.1016/j.molonc.2015.09.002.
