# Supplementary material for: High HER2 protein levels correlate with increased survival in breast cancer patients treated with anti‐HER2 therapy
Source: Mol Oncol. 2015 Sep 15;10(1):138–47. doi: 10.1016/j.molonc.2015.09.002 (PMC4968773; doi:10.1016/j.molonc.2015.09.002)
Supplement: Supplementary file 1 — Supplementary data [file MOL2-10-138-s001.doc]

**Supplementary Table 1.** Breast cancer cell lines used in study.

| **Cell Line** | **Study Sample ID** | **HER2 IHC** | **HER2 FISH** | **Her2 amol/µg** |
| --- | --- | --- | --- | --- |
| **MDA231** | ctrl_1 | +0 | no ampl | 0.0 |
| **MCF7** | ctrl_2 | +0 | no ampl | 0.0 |
| **T47D** | ctrl_3 | +2 | no ampl | 697.0 |
| **ZR75-1** | ctrl_4 | +2 | no ampl | 988.0 |
| **SKBR3** | ctrl_5 | +3 | ampl | 7717.0 |
| **HCC1954** | ctrl_6 | +3 | ampl | 10020.0 |
| **ZR 75-30** | ctrl_7 | +3 | ampl | 16964.0 |

**Supplementary Table 2.** Clinicopathological characteristics of patients included in the survival analysis.

| **Characteristics** | **Nº** | **%** |
| --- | --- | --- |
| **Histological grade**  G1-G2  G3 | 33  62 | 35  65 |
| **Pathological stage T**  Tx-T1  T2-T4  Unknown | 50  42  3 | 53  44  3 |
| **Pathological stage N**  Nx-N0  N1-N3  Unknown | 40  52  3 | 42  55  3 |
| **Hormone receptor status (HR)**  Negative  Positive | 27  68 | 28  72 |
| **HER2 amplification by central ISH**  No amplification  Amplification  Not determined | 6  87  2 | 6  92  2 |
| **HER2/CEP17 ratio by central ISH**  <2  2-6.3  ≥6.4  Not determined | 6  32  55  2 | 6  34  58  2 |
| **Pattern of amplification**  Double Minutes  Homogeneous stained regions  Mixed  Not determined | 32  37  18  8 | 34  39  19  8 |
| **Anti-HER2 treatment**  Trastuzumab (single agent)  Trastuzumab + lapatinib  Trastuzumab + pertuzumab  T-DM1 | 71  7  15  2 | 75  7  16  2 |
| **Anti-HER2 treatment setting**  Adjuvant  Metastatic | 68  27 | 72  28 |
| **Disease/Progression-free survival, years**  Adjuvant, Mean (95% CI)  Metastatic, Mean (95% CI) | 3.2 (2.8–3.6)  5.2 (3.5-6.8) |  |
| **Overall survival, years**  Adjuvant, Mean (95% CI)  Metastatic, Mean (95% CI) | 3.1 (2.7-3.4)  4.4 (3.3-5.3) |  |

**Supplementary Table 3.** HER2 expression as measured by SRM-MS according to the different immunohistochemistry (IHC) scores.

SD, standard deviation. ^1^Kruskal-Wallis rank sum test.

|  |  | **Her2_amolµg** | | | | | |
| --- | --- | --- | --- | --- | --- | --- | --- |
|  |  | **Mean** | **SD** | **Min** | **Max** | **N total** | **P-value^1^** |
| HER2 IHC | **+0** | 189.1 | 324.2 | 0.0 | 1348.7 | 41 | **<0.001** |
|  | **+1** | 259.9 | 357.2 | 0.0 | 1748.0 | 49 |  |
|  | **+2** | 406.7 | 481.9 | 0.0 | 2731.5 | 51 |  |
|  | **+3** | 4214.1 | 3768.0 | 163.7 | 17446.7 | 136 |  |

**Supplementary Table 4.** HER2 expression as measured by SRM-MS according to the HER2 gene amplification status by in situ hybridization (ISH).

No ampl, absence of amplification; Ampl, presence of amplification; SD, standard deviation; ^1^Wilcoxon test.

|  | | **Her2 (amol/µg)** | | | | | |
| --- | --- | --- | --- | --- | --- | --- | --- |
|  | | **Mean** | **SD** | **Min** | **Max** | **N total** | **P-value^1^** |
| HER2 ISH | **No ampl** | 383.8 | 339.1 | 0.0 | 1748.0 | 60 | **<0.001** |
|  | **Ampl** | 4151.2 | 3682.1 | 272.8 | 17446.7 | 124 |  |
|  |  | | | | | | |

**Supplementary Table 5.** Average HER2 levels as detected by SRM-MS according to sample collection year by IHC category (cat)

SD, Standard deviation; *P* value calculated with ANOVA.

|  | | |  | **Her2_amolµg** | | |
| --- | --- | --- | --- | --- | --- | --- |
| **IHC cat** | | **Sample year** | **n** | **Average** | **SD** | ***P* value** |
| **+0** | <2008 | | 2 | 365,25 | 516,54 | .22 |
|  | 2008 | | 5 | 232,76 | 323,61 |  |
|  | 2009 | | 15 | 131,21 | 226,17 |  |
|  | 2010 | | 11 | 353,70 | 457,82 |  |
|  | 2011 | | 6 | ,00 | ,00 |  |
| **+1** | <2008 | | 3 | 174,00 | 301,38 | .17 |
|  | 2008 | | 3 | 424,68 | 391,54 |  |
|  | 2009 | | 9 | 375,90 | 289,88 |  |
|  | 2010 | | 23 | 310,03 | 425,25 |  |
|  | 2011 | | 11 | 38,63 | 128,11 |  |
| **+2** | 2008 | | 4 | 534,75 | 392,33 | .31 |
|  | 2009 | | 16 | 245,58 | 181,15 |  |
|  | 2010 | | 18 | 555,78 | 713,87 |  |
|  | 2011 | | 5 | 194,01 | 265,66 |  |
|  | 2012-13 | | 6 | 336,28 | 177,04 |  |
| **+3** | <2008 | | 25 | 3820,55 | 3366,02 | .40 |
|  | 2008 | | 21 | 4570,58 | 3071,53 |  |
|  | 2009 | | 25 | 5155,53 | 4751,08 |  |
|  | 2010 | | 19 | 2879,73 | 2523,23 |  |
|  | 2011 | | 15 | 3932,04 | 3646,72 |  |
|  | 2012-13 | | 28 | 3726,41 | 3520,68 |  |

**Supplementary Table 6.** Details of discordant cases between SRM-MS and IHC/ISH after central ISH retest.

| **Oncoplex ID** | **HER2 amolµg** | **SRM-MS cat** | **HER2 IHC Local** | **HER2 status Local** | **HER2 status Central** | **Amplification pattern** | **PTN** | **Specimen** | **Diagnosis** | **Grade** | **HR** |
| --- | --- | --- | --- | --- | --- | --- | --- | --- | --- | --- | --- |
| **D0606** | 349.8 | Negative | Equivocal | Positive | **Positive** | DM | T1N0 | SR | IDC | 3 | POS |
| **D0595** | 435.3 | Negative | Equivocal | Positive | **Positive** | DM | T2N1 | SR | IDC | 2 | POS |
| **D0596** | 478.0 | Negative | Equivocal | Positive | **Positive** | DM | T1N0 | SR | IDC | 3 | NEG |
| **C0593** | 163.8 | Negative | Positive | Positive | Negative | - | T4NX | SR | IDC | 2 | NEG |
| **D0188** | 272.8 | Negative | Positive | Positive | **Positive** | DM | T2N1 | SR | IDC | 3 | POS |
| **C0568** | 283.3 | Negative | Positive | Positive | Negative | - | T2N1 | SR | IDC | 3 | POS |
| **D0152** | 287.8 | Negative | Positive | Positive | **Positive** | MIX | T1N0 | SR | IDC | 3 | NEG |
| **D0156** | 382.4 | Negative | Positive | Positive | **Positive** | DM | T1N0 | SR | IDC | 1 | POS |
| **D0665** | 424.9 | Negative | Positive | Positive | Negative | - | T1N1 | SR | TC | 1 | POS |
| **D0656** | 443.6 | Negative | Positive | Positive | **Positive** | DM | T2N0 | SR | IDC | 2 | POS |
| **A0090** | 500.0 | Negative | Positive | Positive | Negative | - | T1N1 | SR | IDC | 3 | POS |
| **C0594** | 522.8 | Negative | Positive | Positive | Negative | - | TXN2 | RD | IDC | 3 | NEG |
| **D0165** | 535.3 | Negative | Positive | Positive | **Positive** | DM | T3N3 | RD | IDC | 3 | NEG |
| **D0177** | 542.8 | Negative | Positive | Positive | **Positive** | DM | T1N0 | SR | IDC | 2 | POS |
| **A0097** | 550.0 | Negative | Positive | Positive | Negative | - | T2N1 | SR | IDC | 3 | POS |
| **A0091** | 580.0 | Negative | Positive | Positive | Negative | - | T1N2 | SR | IDC | 2 | POS |
| **D0187** | 639.8 | Negative | Positive | Positive | **Positive** | MIX | T2N0 | SR | IDC | 3 | POS |
| **D0574** | 1348.7 | Positive | Negative | Negative | **Negative** | - | TXN0 | SR | IDC | xx | POS |
| **D0503** | 771.3 | Positive | Negative | Negative | **Negative** | - | T1N0 | SR | IDC | 2 | POS |
| **D0547** | 834.5 | Positive | Negative | Negative | **Negative** | - | T2N1 | SR | IDC | 3 | POS |
| **D0569** | 1748.0 | Positive | Negative | Negative | **Negative** | - | T1N0 | SR | PC | 3 | POS |
| **Ctrl_4** | 988.0 | Positive | Equivocal | Negative**^1^** | **Negative^1^** | - | xx | xx | xx | xx | xx |
| **D0664** | 764.3 | Positive | Positive | Positive | **Negative** | - | T4N2 | SR | IDC | 3 | POS |
| **D0173** | 959.8 | Positive | Positive | Positive | **Negative** | - | T1N0 | SR | IDC | 2 | POS |

SRM-MS cat: Negative, <740 amolµg; Positive, >740 amolµg. HER2 status local: Positive, IHC positive (3+) or ISH amplified (IHC equivocal). HER2 status central: Positive, ISH amplified; Negative, ISH non amplified. In **bold** discordant cases between SRM-MS and ISH after central re-test. DM, double minutes; MIX, mixed pattern. SR, surgical resection; RD, recurrent disease. IDC, invasive ductal carcinoma; HR, Hormone receptors. POS, positive; NEG, negative. ^1^results based on literature data.

**Supplementary Table 7.** HER2 expression levels by SRM, HER2 gene copy number (GCN) and HER2/CEP17 ratio in ISH-positive breast cancer.

SD, standard deviation. ZR 75-30 cell line was not included in the analysis as central ISH re-test was not available.

|  | **N** | **Mean** | **SD** | **Min** | **Max** |
| --- | --- | --- | --- | --- | --- |
| **Her2 amolµg** | 123 | 4047.1 | 3508.9 | 272.8 | 17446.7 |
| **HER2 GCN** | 123 | 14.0 | 4.2 | 5.2 | 22.2 |
| **HER2/CEP17 Ratio** | 123 | 7.2 | 2.5 | 2.1 | 15.0 |

**Supplementary Table 8.** HER2 expression levels by SRM-MS according to amplification pattern.

DM, double minutes; HSR, homogeneously stained regions; MIX, mixed pattern. SD, standard deviation. ^1^Kruskal-Wallis rank sum test.

|  | | **Her2 (amol/µg)** | | | | | |
| --- | --- | --- | --- | --- | --- | --- | --- |
|  | | **Mean** | **SD** | **Min** | **Max** | **N total** | **P-value^1^** |
| Pattern of amplification | **DM** | 2176.4 | 1908.1 | 272.8 | 8070.0 | 46 | **<0.001** |
|  | **HSR** | 5462.9 | 3368.4 | 1099.3 | 17446.7 | 50 |  |
|  | **MIX** | 4612.1 | 4439.6 | 287.8 | 16303.3 | 27 |  |

**Supplementary Table 9.** Distribution for pattern of HER2 amplification in SRM-MS negative (<740) and positive (>740) tumors.

DM, double minutes; HSR, homogeneously stained regions; MIX, mixed pattern.

|  |  | **DM** | **MIX** | **HSR** |  |
| --- | --- | --- | --- | --- | --- |
| **HER2 (amol/µg** | <740 | 8 | 2 | 0 |  |
|  | >740 | 38 | 25 | 50 |  |
|  | **Total** | 46 | 27 | 50 | 123 |

**Supplementary Table 10.** ROC analysis.

The optimal cutoff for HER2 levels by SRM-MS, HER2/CEP17 ratio and HER2 GCN discriminating between a positive or negative result, in terms of response to treatment and prolonged survival, was determined using ROC analysis. Patients were dichotomized into good responders (absence of disease progression at last follow up) or poor responders (presence of disease progression at last follow up) and into good survival (alive at last follow up) versus poor survival (dead at last follow up) groups. The cutoff values of HER2 amol/µg of 2200, HER2/CEP17 ratio of 6.4 and HER2 GCN of 12.50 were chosen as the optimal cutoff values for survival analyses. The HER SRM of 2200 amol/µg threshold represents an intermediate value between optimal thresholds distinguishing good versus poor survival (2110.25 amol/µg) and good versus poor responders (2302.25 amol/µg) in the adjuvant population. The sensitivity and specificity of this threshold in predicting survival was 100% and 65.57% in the adjuvant setting, and 56.25% and 100% in the metastatic setting. AUC: Area under ROC curve.

| **Oncoplex ID** | **HER2 amolµg** | **SRM-MS cat** | **HER2 IHC Local** | **HER2 status Local** | **HER2 status Central** | **Amplification pattern** | **PTN** | **Vital status** | **Specimen** | **Diagnosis** | **Grade** | **HR** |
| --- | --- | --- | --- | --- | --- | --- | --- | --- | --- | --- | --- | --- |
| **D0606** | 349.8 | Negative | Equivocal | Positive | **Positive** | DM | T1N0 | - | SR | IDC | 3 | POS |
| **D0595** | 435.3 | Negative | Equivocal | Positive | **Positive** | DM | T2N1 | - | SR | IDC | 2 | POS |
| **D0596** | 478.0 | Negative | Equivocal | Positive | **Positive** | DM | T1N0 | ALIVE | SR | IDC | 3 | NEG |
| **C0593** | 163.8 | Negative | Positive | Positive | Negative | - | T4NX | DEAD | SR | IDC | 2 | NEG |
| **D0188** | 272.8 | Negative | Positive | Positive | **Positive** | DM | T2N1 | - | SR | IDC | 3 | POS |
| **C0568** | 283.3 | Negative | Positive | Positive | Negative | - | T2N1 | - | SR | IDC | 3 | POS |
| **D0152** | 287.8 | Negative | Positive | Positive | **Positive** | MIX | T1N0 | - | SR | IDC | 3 | NEG |
| **D0156** | 382.4 | Negative | Positive | Positive | **Positive** | DM | T1N0 | ALIVE | SR | IDC | 1 | POS |
| **D0665** | 424.9 | Negative | Positive | Positive | Negative | - | T1N1 | - | SR | TC | 1 | POS |
| **D0656** | 443.6 | Negative | Positive | Positive | **Positive** | DM | T2N0 | ALIVE | SR | IDC | 2 | POS |
| **A0090** | 500.0 | Negative | Positive | Positive | Negative | - | T1N1 | DEAD | SR | IDC | 3 | POS |
| **C0594** | 522.8 | Negative | Positive | Positive | Negative | - | TXN2 | DEAD | RD | IDC | 3 | NEG |
| **D0165** | 535.3 | Negative | Positive | Positive | **Positive** | DM | T3N3 | DEAD | RD | IDC | 3 | NEG |
| **D0177** | 542.8 | Negative | Positive | Positive | **Positive** | DM | T1N0 | ALIVE | SR | IDC | 2 | POS |
| **A0097** | 550.0 | Negative | Positive | Positive | Negative | - | T2N1 | DEAD | SR | IDC | 3 | POS |
| **A0091** | 580.0 | Negative | Positive | Positive | Negative | - | T1N2 | DEAD | SR | IDC | 2 | POS |
| **D0187** | 639.8 | Negative | Positive | Positive | **Positive** | MIX | T2N0 | ALIVE | SR | IDC | 3 | POS |
| **D0574** | 1348.7 | Positive | Negative | Negative | **Negative** | - | TXN0 | - | SR | IDC | xx | POS |
| **D0503** | 771.3 | Positive | Negative | Negative | **Negative** | - | T1N0 | - | SR | IDC | 2 | POS |
| **D0547** | 834.5 | Positive | Negative | Negative | **Negative** | - | T2N1 | - | SR | IDC | 3 | POS |
| **D0569** | 1748.0 | Positive | Negative | Negative | **Negative** | - | T1N0 | - | SR | PC | 3 | POS |
| **Ctrl_4** | 988.0 | Positive | Equivocal | Negative**^1^** | **Negative^1^** | - | xx | xx | xx | xx | xx | xx |
| **D0664** | 764.3 | Positive | Positive | Positive | **Negative** | - | T4N2 | DEAD | SR | IDC | 3 | POS |
| **D0173** | 959.8 | Positive | Positive | Positive | **Negative** | - | T1N0 | ALIVE | SR | IDC | 2 | POS |

**Supplementary Table 11.** DFS and OS HRs comparison using two different cut points (740 and 2200).

| **Adjuvant (n=68)** | | | **HR** | **CI_95%_** | ***p-value^1^*** |
| --- | --- | --- | --- | --- | --- |
| **DFS** | | **740** | 0.705 | 0.09-5.514 | 0.738 |
|  |  | **2200** | 0.219 | 0.059 - 0.811 | 0.0125 |
|  |  | |  |  |  |
| **OS** | | **740** | 0.274 | 0.031-2.375 | 0.209 |
|  |  | **2200** | NA | NA - NA | 0.0014 |
| **Metastatic (n=27)** | | | **HR** | **CI_95%_** | ***p-value^1^*** |
| **DFS** | | **740** | 0.331 | 0.104-1.056 | 0.05 |
|  |  | **2200** | 0.561 | 0.243-1.294 | 0.169 |
|  |  | |  |  |  |
| **OS** | | **740** | 0.183 | 0.054-0.623 | 0.002 |
|  |  | **2200** | 0.198 | 0.069 - 0.566 | 0.0008 |

NA, Not assessable; HR, hazard ratio; CI, confidence interval.

**Supplementary Table 12.** Correlation between HER2 status by SRM and ISH and clinicopathological characteristics in the adjuvant series.

^1^Chi square test; ^2^Fisher exact test; ^3^Wilcoxon test.

|  | | **HER2 (amol/ug)** | | | **HER2 Ratio** | | | **HER2 GCN** | | |
| --- | --- | --- | --- | --- | --- | --- | --- | --- | --- | --- |
|  |  | **< 2200**  **(n=28, 41.2%)** | **>= 2200**  **(n=40, 58.8%)** | ***p-value*** | **< 6.4**  **(n=22, 33.3%)** | **>= 6.4**  **(n=44, 66.7%)** | ***p-value*** | **< 12.5**  **(n=30, 45.4%)** | **>= 12.5**  **(n=36, 54.6%)** | ***p-value*** |
| **HR** | **Negative** | 7 (10.3%) | 10 (14.7%) | *0.968^1^* | 7 (10.6%) | 10 (15.2%) | *0.619^1^* | 8 (12.1%)) | 9 (13.6%) | *0.898^1^* |
|  | **Positive** | 21 (30.89%) | 30 (44.1%) |  | 15 (22.7%) | 34 (51.5%) |  | 22 (33.3%) | 27 (40.9%) |  |
| **Grade** | **G1** | 1 (1.5%) | 0 (0%) | ***0.023^2^*** | 1 (1.5%) | 0 (0%) | *0.208^2^* | 1 (1.5%) | 0 (0%) | *0.695^2^* |
|  | **G2** | 15 (22.1%) | 11 (16.2%) |  | 10 (22.7%) | 15 (16.7%) |  | 12 (18.2%) | 13 (19.7%) |  |
|  | **G3-G4** | 12 (17.7%) | 29 (42.7%) |  | 11 (18.2%) | 29 (43.9%) |  | 17 (25.8%) | 23 (34.9%) |  |
| **Pahological T** | **TX - T1** | 14 (20.6%) | 28 (41.2%) | *0.157^1^* | 14 (21.2) | 26 (39.4) | *0.929^1^* | 17 (25.8%) | *23 (34.9%)* | *0.730^1^* |
|  | **T2 - 4** | 14 (20.6%) | 12 (17.7%) |  | 8 (12.1%) | 18 (27.3%) |  | *13 (19.7%)* | *13 (19.7%)* |  |
| **Pathological N** | **NX – N0** | 15 (22.1%) | 20 (29.4%) | *0.965^1^* | *12 (18.2%)* | *22 (33.3%)* | *0.931^1^* | *20 (30.3%)* | *14 (21.2%)* | ***0.045****^1^* |
|  | **N1 – N3** | 13 (19.1%) | 20 (29.4%) |  | *10 (15.2%)* | *22 (33.3%)* |  | 10 (15.2%) | *22 (33.3%)* |  |
| **Follow up, Years**  **Mean (sd)** | | 3.5 (1.5) | 3.4 (1.5) | *0.699^3^* | 3.5 (1.4) | 3.3 (1.5) | *0.414^3^* | 3.3 (1.5) | 3.4 (1.4) | *0.918^3^* |
| **Disease progression** | **No** | 19 (27.9%) | 37 (54.4%) | ***0.021****^2^* | 16 (24.2%) | 38 (57.6%) | *0.310^1^* | 24 (36.4%) | 30 (45.5%) | *0.977^1^* |
|  | **Yes** | 9 (13.2%) | 3 (4.4%) |  | 6 (9.1%) | 6 (9.01%) |  | 6 (9.1%) | 6 (9.1%) |  |
| **Vital status** | **Dead** | 7 (10.3%) | 0 (0%) | ***0.001****^2^* | 4 (6.1%) | 3 (4.6%) | *0.210^2^* | 4 (6.1%) | 3 (4.6%) | *0.693^2^* |
|  | **Alive** | 21 (30.9%) | 40 (58.8%) |  | 18 (27.3%) | 41 (62.1%) |  | 26 (39.4%) | 33 (50%) |  |

**Supplementary Table 13.** Correlation between HER2 status by SRM and ISH and clinicopathological characteristics in metastatic series.

|  | | **HER2 (amol/ug)** | | | **HER2 Ratio** | | | **HER2 GCN** | | |
| --- | --- | --- | --- | --- | --- | --- | --- | --- | --- | --- |
|  |  | **< 2200**  **(n=9, 33.3%)** | **>= 2200**  **(n=18, 66.7%)** | ***p-value*** | **< 6.4**  **(n=16, 59.3%)** | **>= 6.4**  **(n=11, 40.7%)** | ***p-value*** | **< 12.5**  **(n=13, 48.1%)** | **>= 12.5**  **(n=14, 51.9%)** | ***p-value*** |
| **Follow up, Years** | **Mean (sd)** | 6.0 (4.7) | 8.5 (5.5) | *0.232^3^* | 6.2 (4.3) | 9.8 (6.0) | *0.056^3^* | 5.8 (4.2) | 9.4 (5.7) | *0.105^3^* |
| **Disease progression** | **No** | 0 (0%) | 2 (7.4%) | *0.539^2^* | 1 (3.7%) | 1 (3.7%) | *0.987^2^* | 0 (0%) | 2 (7.4%) | *0.482^2^* |
|  | **Yes** | 9 (33.3%) | 16 (59.3%) |  | 15 (55.6%) | 10 (31.0%) |  | 13 (48.1%) | 12 (44.4%) |  |
| **Vital status** | **Dead** | 9 (33.3%) | 7 (25.9%) | ***0.003^2^*** | 11 (40.7%) | 5 (18.5%) | *0.264^2^* | 11 (40.7%) | 5 (18.5%) | ***0.018^2^*** |
|  | **Alive** | 0 (0%) | 11 (40.7%) |  | 5 (18.5%) | 6 (22.2%) |  | 2 (7.4%) | 9 (33.3%) |  |

^1^Chi square test; ^2^Fisher exact test; ^3^Wilcoxon test.

**Supplementary Table 14.** Disease free survival and overall survival hazard ratios according to HER2 pattern of amplification. DM, doble minutes, HSR, homogeneously stained regions; MIX, mixed; HR, hazard ratio; CI, confidence interval.

| **Adjuvant (n=63)** | | **HR** | **CI_95_** | ***p-value*** |
| --- | --- | --- | --- | --- |
| **DFS** | DM/HSR | 1.59 | 0.35-7.14 | 0.546 |
|  | DM/MIX | 0.83 | 0.18-3.70 | 0.804 |
|  | HSR/MIX | 0.52 | 0.10-2.60 | 0.427 |
| **OS** | DM/HSR | 2.62 | 0.23-29.80 | 0.438 |
|  | DM/MIX | 0.96 | 0.10-8.93 | 0.973 |
|  | HSR/MIX | 0.37 | 0.03-4.44 | 0.431 |
| **Metastatic (n=24)** | | **HR** | **CI_95_** | ***p-value*** |
| **DFS** | DM/HSR | 0.50 | 0.13-1.85 | 0.297 |
|  | DM/MIX | 1.13 | 0.21-6.01 | 0.883 |
|  | HSR/MIX | 2.28 | 0.39-13.42 | 0.362 |
| **OS** | DM/HSR | 4.14 | 0.89-8.41 | 0.067 |
|  | DM/MIX | 5.63 | 0.71-44.45 | 0.101 |
|  | HSR/MIX | 1.36 | 0.12-15.56 | 0.804 |
